# Application of *Bacillus tequilensis* for the control of gray mold caused by *Botrytis cinerea* in blueberry and mechanisms of action: inducing phenylpropanoid pathway metabolism

**DOI:** 10.3389/fmicb.2024.1455008

**Published:** 2024-08-30

**Authors:** Qianjie Du, Raoyong Li, Li Liu, Lin Chen, Junrong Tang, Jia Deng, Fang Wang

**Affiliations:** ^1^Key Laboratory of National Forestry and Grassland Administration on Biodiversity Conservation in Southwest China, Southwest Forestry University, Kunming, China; ^2^Forestry College, Southwest Forestry University, Kunming, China; ^3^Key Laboratory for Forest Resources Conservation and Utilization in the Southwest Mountains of China, Ministry of Education, Southwest Forestry University, Kunming, China

**Keywords:** *Bacillus tequilensis*, blueberry, induced resistance, phenylpropanoids, postharvest disease, biological control

## Abstract

**Background:**

*Botrytis cinerea* a blueberry gray mold, is one of the main diseases affecting postharvest storage, causing significant losses. Several studies have shown that *Bacillus tequilensis* can prevent the growth of plant pathogens by producing various antibacterial substances, and can induce plant resistance. However, research on the biological management of post-harvest gray mold in blueberries using *B. tequilensis* remains unclear.

**Methods:**

To better control the postharvest gray mold of blueberry, the effects of *B. tequilensis* KXF6501 fermentation solution (YY) and KXF6501 cell-free supernatant (SQ) on the induction of disease resistance in blueberry fruits were studied using biochemical and transcriptomic analyses.

**Results:**

We found that YY controlled the conidial germination and mycelial growth of *B. cinerea in vitro*, followed by SQ. After 3 d of culture, the lesion diameter and incidence of gray mold in blueberry fruits inoculated with YY and SQ were smaller than those in the control group. Therefore, gray mold in blueberries was effectively controlled during the prevention period, and the control effect of YY was better than that of SQ. Transcription spectrum analysis of blueberry peel tissue showed that the YY- and SQ-induced phenylpropane metabolic pathways had more differentially expressed genes (DEGs) than other biological pathways. In addition, biochemical analyses showed that YY treatment effectively enhanced the activity of enzymes related to the phenylpropane pathway (phenylalanine ammonialyase [PAL], cinnamate 4-hydroxylase [C4H], 4-coumarate CoA ligase [4CL], and polyphenol oxidase [PPO]) and stimulated the synthesis of lignin, total phenols, and flavonoids, followed by SQ. Compared with the control, the YY and SQ treatments reduced the weight loss rate and better maintained the appearance and nutritional quality of the blueberry fruits.

**Conclusion:**

Our findings suggest that *B. tequilensis* KXF6501 is potentially useful as a suitable bio-control agent in harvested blueberries.

## 1 Introduction

Blueberries, classified within the genus *Vaccinium* of the family *Ericaceae* (Bell et al., 2021). Blueberries are known as the “king of berries”, due to their abundance of amino acids, trace elements and its unique compounds with high biological activity (anthocyanins, tannins, flavonoids and phenolic acids, etc.), so it is highly beneficial to the liver, as it helps to prevent cancer, prolong life, and protect the heart (Chen et al., 2015, 2017; Miller et al., 2019; Shi et al., 2019a). Gray mold caused by *Botrytis cinerea* is one of the most important diseases affecting blueberries, limiting the storage, transportation, and sales of blueberries (Rivera et al., 2013; Ji et al., 2019). *B. cinerea* causes fruit tissue death by activating toxin production, degrading enzyme production, oxygen-active substances, and programmed cell death, which seriously affects the economic value of blueberries (Bell et al., 2021; Bi et al., 2023). At present, chemical control is the oldest and most common method of blueberry postharvest storage and is based on the use of chemicals, such as chlorine, chlorine dioxide, sulfur dioxide, and some synthetic fungicides, to reduce postharvest diseases (Bell et al., 2021). Although chemical treatments can effectively control blueberry diseases, they can easily lead to drug residues and environmental pollution (Bell et al., 2021). Therefore, it is of great significance to seek safe and efficient storage and preservation technologies for the postharvest preservation of fruits, vegetables, and human health.

Biological control technologies for antagonistic microorganisms have become a significant focus of research in the field of fruit preservation because of their high efficiency, lack of pesticide residues, and easy degradation (Alvarez et al., 2019; Chen et al., 2020). *Bacillus* spp. strains attracted considerable attention as biological control agents (BCAs) because they produce endogenous spores, enzymes, antibiotics, and toxins (Kiesewalter et al., 2020). Simultaneously, they compete for limited food and space, obviating disease-causing pathogenic fungi from growing in their hosts (Panebianco et al., 2015; Papoutsis et al., 2019). Kurniawan et al. (2018) showed that several *Bacillus* spp. have antifungal properties against *B. cinerea* of blueberries. *Bacillus subtilis* KLBC BS6 was effective in inhibiting *B. cinerea* conidia germination and mycelial growth, remarkably reducing gray mold outbreaks and enhancing the disease-resistant enzymatic activities of phenylalanine ammonia-lyase [PAL], polyphenol oxidase [PPO], Chitinase [CHI], and superoxide dismutase [SOD] in blueberry fruits (Lu et al., 2021). *Bacillus tequilensis* JN-369 induces and improves the SOD, CAT, and POD activities in rice, thus enabling resistance against rice blast infection (Zhou et al., 2021).

Research has shown that *B. tequilensis* can prevent the growth of plant pathogens by producing various antibacterial substances, such as biosurfactants and lipopeptides, and can induce plant resistance (Singh and Sharma, 2020). However, research on the biological management of post-harvest gray mold in blueberries using *B. tequilensis* remains unclear. Therefore, the primary objectives of this study were to (a) evaluate the antimicrobial properties of *B. tequilensis* KXF6501 on *B. cinerea* in post-harvest blueberry fruits, (b) investigate the changes in RNA-Seq transcription patterns in blueberry fruits induced by fermentation solution (YY) and cell-free supernatant (SQ), and (c) explore the impact of YY and SQ in preventive treatments on enzyme activities related to the phenylpropane pathway in blueberry fruits.

## 2 Materials and methods

### 2.1 Fruits and microorganism sources

Jewelry blueberries were hand harvested from a mercantile orchard in Chengjiang, Yuxi, Yunnan Province, China (24°38’32” N latitude, 102°53’28” E longitude). The orchard has superior natural environment, large-scale planting, rich varieties (such as jewelry, Emerald and Lexie) and high-level planting and management technology. Select blueberries that are uniformly mature, evenly sized, free from diseases and pests, and without mechanical damage, and bring them back to the laboratory immediately. Immersed in 2% (v/v) sodium hypochlorite for 30 s and drying at 22°C for disinfection. Subsequently, they were shared among different groups based on a completely randomized block design.

*B. cinerea* was isolated from diseased blueberries, cultured on PDA for 5 d at 25 °C until sporulation occurred. Subsequently, spore suspensions were produced by flooding *B. cinerea* cultures with sterile distilled water, and spore concentrations were controlled to 1 × 10^5^ conidia mL^–1^ using a haemocytometer.

Strawberries that appeared healthy and showed no physical damage were used to isolate the *B. tequilensis* strain KXF6501. The strawberry fruit was soaked in a 250 mL beaker containing 100 mL of PBS buffer for 2 h to prepare a stock solution. Subsequently, 1 mL of the stock solution was diluted to 10^–4^, 10^–5^, and 10^–6^, and 100 μL of each diluted sample was spread onto nutrient agar (NA) plates, respectively. Plates were incubated at 28 ° for 24 h, and a single colony was transferred and purified on NA medium. The isolated *B. tequilensis* strain KXF6501 was identified using the 16S rDNA gene. Meanwhile, it is maintained at −80 °C in a nutrient broth with glycerol’s by the Plant Pathology Laboratory of Southwest Forestry University. The strain was activated and grown for 24 h at 28 °C on nutrient agar.

### 2.2 Preparation of KXF6501 fermentation solution and cell-free supernatant

Culture suspension of KXF6501 (1 × 10^7^ CFU mL^–1^) incubated for 24 h was prepared into the following two kinds of treatment solutions: (1) KXF6501 fermentation solution (YY): culture suspension of KXF6501 (1 × 10^7^ CFU mL^–1^) incubated for 24 h; (2) KXF6501 cell-free supernatant (SQ): culture suspension of KXF6501 (1 × 10^7^ CFU mL^–1^) was incubated for 24 h, filtered by sterile filter membrane with a diameter of 0.22 micron, and the supernatant was collected for later use.

### 2.3 Effects of KXF6501 fermentation solution and cell-free supernatant against *B. cinerea in vitro*

The antagonistic effects of YY and SQ on *B. cinerea* were assessed using the dual-culture plate approach described by Shi et al. (2014). In a plate containing fresh PDA (diameter, 90 mm), a 6 mm diameter plug of a 5-d *B. cinerea* mycelial culture was centered. Then, four pieces of sterile filter paper with a diameter of 6 mm were inoculated with 5 μL treating fluids (YY or SQ) at a distance of 3 cm away from fungal inoculums. PDA inoculated with only the pathogenic fungus served as the control. Both inoculated and controls plates were incubated for 5 d at 25 °C, and colonies were measured and recorded. Each test was performed in triplicate by three plates.

### 2.4 Efficacy of KXF6501 fermentation solution and cell-free supernatant on the *B. cinerea* conidia germination *in vitro*

According to Wang F. et al. (2019), conidial germination and germ tube elongation of *B. cinerea* were measured in potato glucose broth to further assess the antagonistic action of YY and SQ. We mixed 1 mL of a suspension of fungal spores (1 × 10^7^ conidia mL^–1^) with 1 mL of YY or SQ supplemented with 10 mL of PDB in 50 mL Erlenmeyer flasks, and the fungal spore suspension (1 × 10^7^ conidia mL^–1^) alone served as a control (CK). After inoculation, it was cultured in a shaking table (28 °C, 120 rpm/min) for 2 and 4 h, and the germination rate and germ tube lengths were observed using a microscope (a minimum of 100 *B. cinerea* spores per repetition).

2.5 Effects of KXF6501 fermentation solution and cell-free supernatant against blueberry gray mold *in vivo*

The pretreated blueberry fruits were divided into three groups and placed in empty sterile Petri dishes. With a sterile needle, a 3 mm deep wound was made on each blueberry fruits, and each wound was treated with 10 μL of YY, SQ or sterile distilled water (CK). After 2 h, 10 μL of *B. cinerea* spore suspension (1 × 10^5^ Conidia mL^–1^) was inoculated into the same wound (Shi et al., 2019b). After the spore suspension is completely absorbed, fruits were stored at 22 °C and 85–95%. Disease incidence and lesion diameter were observed and recorded daily (Wu et al., 2022; Wang et al., 2024). The lesion diameter was measured using the criss-cross method. The incidence rate was calculated using the following formula: incidence rate (%) = (number of diseased fruits / total number of fruits) × 100%. Each treatment had three independent replicates, with eight blueberries per replicate and 72 blueberries per trial. All the experiments were performed twice.

### 2.6 Transcriptomic analysis of blueberry fruits treated with KXF6501 fermentation solution and cell-free supernatant

#### 2.6.1 Extraction of RNA, RNA sequencing, and identification of DEGs

Blueberry fruits were soaked in YY (YY-1, YY-2, YY-3), SQ (SQ-1, SQ-2, SQ-3), or sterile distilled water (CK-1, CK-2, CK-3). After 24 h of storage, peel samples were frozen in liquid nitrogen and total RNA was extracted using the MirVana miRNA Isolation Kit. The RNA content was measured using a NanoDrop ND-2000 spectrophotometre, and cDNA libraries were created using a TruSeq Stranded mRNA Sample Preparation Kit. PANOMIX Biotech Co., Ltd. (Sichuan, China) conducted a transcriptomic analysis to remove low-quality reads to ensure high-quality data. DEG-seq was used to identify DEGs with thresholds of | log2FC| > 1 and *P*-value < 0.05.

#### 2.6.2 GO and KEGG analysis on DEGs

For gene function annotation, the DEGs were classified using the GO database^1^ and metabolic pathways enriched for DEGs were identified using the KEGG database (KEGG).^2^

#### 2.6.3 RNA-seq verification using real-time quantitative reverse transcription-polymerase chain reaction (RT-qPCR)

Fruit peel RNA was extracted and cDNA was synthesized as described previously (Shi et al., 2019c). To validate the results of our transcriptomic analysis, RT-qPCR was performed using the TIB8600 Real-Time PCR System (Triplex International Biosciences, Xiamen, China) with primers specific for Differentially Expressed Genes (DEGs) identified through bioinformatics analysis (Supplementary Table 1).

### 2.7. Effects of KXF6501 fermentation solution and cell-free supernatant on phenylpropanoid metabolism related enzymes and substances in blueberry fruits

Blueberries were subjected to treatments, with YY, SQ, and sterile distilled water as a control (CK) for 5 min. Subsequently, fruits were air-dried, placed in plastic containers, and stored at 22 °C with 85–95% relative humidity for 10 d. Each treatment was replicated three times, and the test was repeated twice. The effects of YY and SQ on the enzymatic activity of PAL, 4CL, PPO, and C4H, as well as the levels of flavonoid, total phenols, and lignin in fruit samples collected at 0, 2, 4, 6, 8, and 10 d post-treatment were assessed. The PAL activity was determined following the protocol outlined by Zhou et al. (2014), whereas the PPO activity was measured according to the method described by Shi et al. (2018). According to methods described by Deng et al. (2016) for determining the activity of C4H and 4CL. The results are expressed in units per gram of fresh fruit weight. A Folin-Ciocalteu colorimetric approach was used to determine total phenols and flavonoids (Ozgen et al., 2010; Yihui et al., 2018; Lu et al., 2019; Zhang et al., 2019), whereas Toscano et al. (2018) approach determined lignin content of fruits.

### 2.8 Effects of KXF6501 fermentation solution and cell-free supernatant on blueberry quality

To assess the effects of YY and SQ on the quality of blueberries, the fruits were subjected to individual soaking treatments: (1) YY, (2) SQ, or (3) sterile distilled water (CK) for 5 min. Subsequently, the fruits were placed in plastic containers and stored at 22 °C with a relative humidity of 85–95% for 10 d. Fruit samples (0, 2, 4, 6, 8, and 10 d) were collected after treatment.

Each 2-d weight of the fruit (in grams) was measured, and the percent loss in weight was computed by applying the formula (weight loss rate % = (A–B)/A), where A is the initial weight of the fruit before treatment, and B is the weight of the fruit at a specific time point. The levels of total soluble solids and reducing sugars in fruits were quantified using a portable refractometer and the anthrone reagent method, while the ascorbic acid and titratable acidity content were determined using 2,6-dichlorophenol indophenol and titration with 0.1 M NaOH, respectively. The color characteristics (L, a, b, c, and h_0_) of the fruits were assessed using a colorimeter.

### 2.9 Statistical approach

Data analysis was conducted using Tukey’s multiple range tests in SPSS22.0 software (IBM Company, Armonk, NY, USA), with significance observed at *P* < 0.05.

## 3 Results

### 3.1 The effect of KXF6501 fermentation solution and cell-free supernatant against *B. cinerea in vitro*

As shown in Table 1, the effects of YY and SQ on the germ tube length and conidial germination rate of *B. cinerea in vitro* were showed. Following the application of YY and SQ, a significant decrease (*P* < 0.05) in the conidial germination rate of *B. cinerea* was noticed in the YY treatment group after 2 h, whereas SQ initially reduced the germination rate of the conidia, and the inhibitory effect gradually decreases over time. For germ tube length, no significant differences were observed between the treatment and control group at the 2-h. However, after 4 h of treatment, the length of *B. cinerea* germinal tubes in the YY group reduced to 51.58 μm, which was noticeably lower compared to both the control group (79.63 μm) and the SQ group (52.60 μm) (*P* < 0.05).

**TABLE 1:** Effects of KXF6501 fermentation solution and cell-free supernatant on germ tube lengths and conidia germination rate of *B. cinerea* grown on PDB over 2 and 4 h.

Treatment	Conidia germination (%)		Germ tube length (μ m)	
	**2 h**	**4 h**	**2 h**	**4 h**
CK	54.00 ± 6.00a	80.33 ± 2.33a	37.09 ± 3.25a	79.63 ± 4.02a
YY	5.33 ± 1.76b	43.67 ± 1.45b	28.63 ± 2.67a	51.58 ± 0.46b
SQ	41.67 ± 8.37a	79.67 ± 1.86a	32.02 ± 1.87a	52.60 ± 2.58b

Based on Tukey’s multiple range test, there were significant (*P* < 0.05) differences between all variables, followed by letters. All data are reported as the mean of three distinct values (standard error).

**FIGURE 1:**
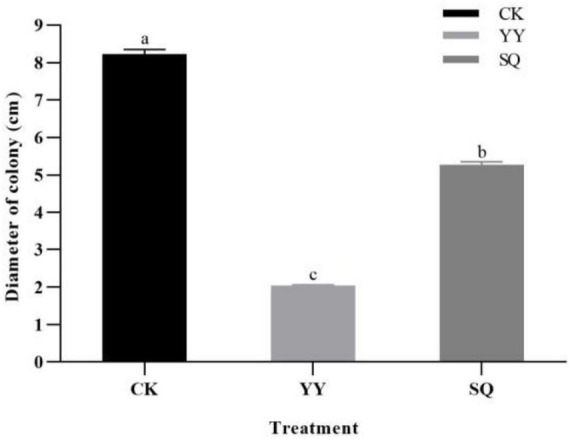
Inhibitory effect of KXF6501 fermentation solution and cell-free supernatant on *B. cinerea* after 5 d. Based on the Tukey’s multiple range test, there are significant (*P* < 0.05) differences between all variables followed by letters. All units are reported as means of three distinct values ± SE. Different lowercase letters indicate significant (*P* < 0.05) differences between treatments by the Tukey’s multiple range test.

The dual-culture plate result indicated that YY demonstrated antagonistic effects on *B. cinerea* mycelial growth, followed by SQ (Supplementary Figure 1A). Following a 5-d incubation period, the colony diameters of the YY and SQ groups measured 2.03 and 5.27 cm, respectively (Figure 1). These values were noticeably lower than those in the control fruits, with inhibition rates of 75.30% and 36.03% (*P* < 0.05), respectively.

### 3.2 Efficacy of KXF6501 fermentation solution and cell-free supernatant in controlling blueberry gray mold *in vivo*

As shown in Table 2, the application of YY reduced gray mold decay attributed to *B. cinerea*, followed by SQ (Supplementary Figure 1B). Following 2-d inoculation, the incidence rate of the YY treatment group was notably the lowest at 13.33%, a statistically significant decrease (*P* < 0.05) compared to that of the control fruits, with the SQ treatment fruits at 20.00%. In the YY and SQ treatment groups, morbidity decreased (*P* < 0.05) by 40% and 20%, respectively, on the 3rd day, compared to that in the control fruits (100%). After 3 d, the lesion diameters in the three groups of infected blueberries exhibited noticeable variation. Blueberries treated with YY after storage for 3, 4, and 5 d had lesion diameters of 0.63, 0.83, and 0.97 cm, respectively, which were notably smaller than those of the SQ treatment group (0.76, 0.91, and 1.07 cm) and the control group (0.96, 1.34, and 1.50 cm) (*P* < 0.05).

**TABLE 2:** The effect of KXF6501 fermentation solution and cell-free supernatant on lesion diameter and disease incidence in blueberries infected with *B. cinerea*.

Treatment	Time after inoculation (d)					
	**1**	**2**	**3**	**3**	**4**	**5**
	**Disease incidence (%)**			**Lesion diameter (cm)**		
CK	13.33 ± 6.67a	33.33 ± 6.67a	100.00 ± 0.00a	0.96 ± 0.01a	1.34 ± 0.01a	1.50 ± 0.02a
YY	0.00 ± 0.00a	13.33 ± 6.67b	60.00 ± 0.00c	0.63 ± 0.02c	0.83 ± 0.03c	0.97 ± 0.02c
SQ	0.00 ± 0.00a	20.00 ± 6.67ab	80.00 ± 0.00b	0.76 ± 0.02b	0.91 ± 0.03b	1.07 ± 0.03b

Based on Tukey’s multiple range test, there were significant (*P* < 0.05) differences between all variables, followed by letters. All data were reported as the mean of three distinct values (SE).

### 3.3 Transcriptional analyses of blueberry fruits treated with KXF6501 fermentation solution and cell-free supernatant

#### 3.3.1 Analysis of biological pathway enrichment and differential expression

**FIGURE 2:**
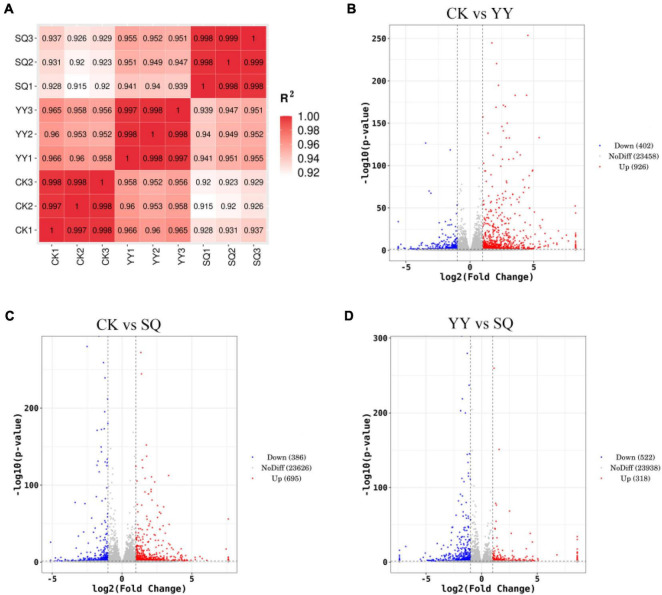
Sample correlation test chart **(A)**, volcano map distribution of DEGs in CK vs. YY **(B)**, CK vs. SQ **(C)**, and YY vs. SQ **(D)**.

In the current study, the R^2^ values between biological replicates ranged 0.92–1.00, indicating the reliability of the transcriptome data (Figure 2A). A comparative analysis was conducted on three sets of sequencing data to elucidate the variations in gene expression among fruits subjected to the YY, SQ, and CK treatments during storage: CK vs. YY, CK vs. SQ, and YY vs. SQ. Differentially expressed genes were identified and assessed based on their expression levels (FPKM) for each gene |log2FC| > 1, *P*-value < 0.05). As illustrated in Figure 2B, 1328 DEGs were detected in the CK vs. YY comparison, with 926 DEGs showing upregulation and 402 DEGs showing downregulation. A total of 1081 DEGs were detected in the comparison between CK and SQ, with 695 upregulated and 386 downregulated genes (Figure 2C). In total, 840 DEGs were identified in the comparison between YY and SQ, with 318 upregulated and 522 downregulated DEGs (Figure 2D). More DEGs were detected in the CK vs. YY comparison group, suggesting a greater difference in quality between blueberries treated with CK and YY. At the same time, volcano plots can visually represent each DEG’s distribution and expression. The differentially expressed genes observed in the comparison between the CK and YY groups exhibited a pronounced level of divergence, suggesting more conspicuous variation among the genes (Figure 2B). The disparity in the quantity and distribution of DEGs in the volcano plot indicates a greater distinction between the blueberries in the CK and YY groups.

**FIGURE 3:**
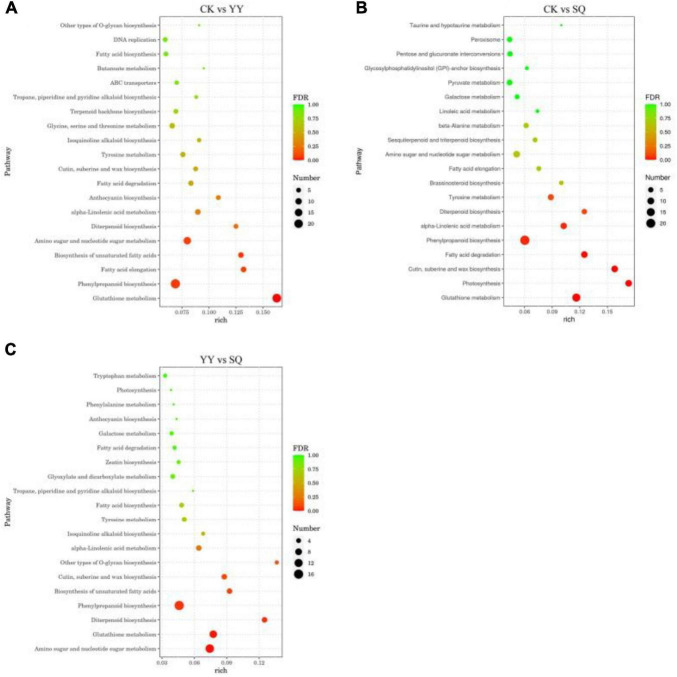
Scatter diagram of DEGS biological pathway enrichment in CK vs YY **(A)**, CK vs SQ **(B)** and YY vs SQ **(C)** fruits.

Among the 1328, 1081, and 840 DEGs of blueberry fruits in the CK vs. YY, CK vs. SQ, and YY vs. SQ groups, 359, 286, and 199 DEGs with pathway annotations were distributed across 93, 86, and 76 pathways, respectively. The top 20 enriched biological pathways in the CK vs. YY, CK vs. SQ, and YY vs. SQ blueberry fruits are depicted in Figure 3. Of these 20 pathways, “phenylpropanoid biosynthesis” emerged as the most important enrichment pathway in the three groups, and it had a small FDR compared with the other pathways.

#### 3.3.2 Transcriptome analyzed and identified consistently DEGs by treated with KXF6501 fermentation solution and cell-free supernatant

**FIGURE 4:**
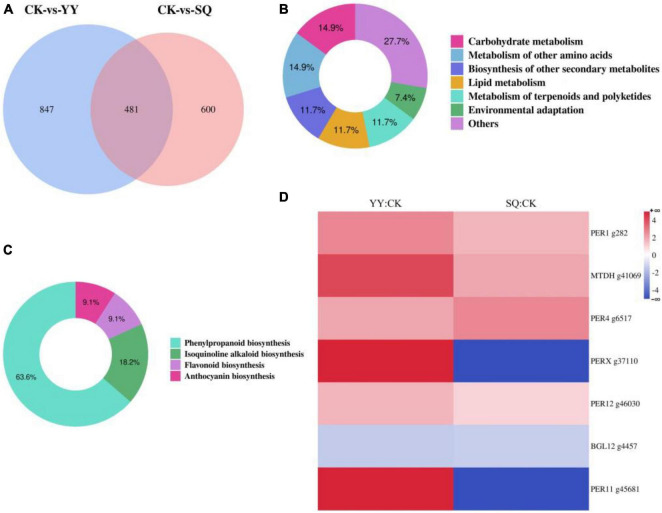
The consistently differentially expressed genes (DEGs) identified through transcriptome analysis in fruits treated with YY and SQ. **(A)** Venn diagram illustrating the non-overlap or overlap of DEGs between the two treatments; **(B)** Categorizes the consistently DEGs in both treatments according to biological pathways; **(C)** A KEGG pathway-based classification of DEGs involved in secondary metabolic biosynthesis in both treatments; **(D)** A cluster analysis of phenylpropanoid biosynthesis-related DEGs expression profiles in both treatments YY and SQ.

Two groups of DEGs (CK vs. YY and CK vs. SQ) were compared to further investigate the alterations in gene expression within the blueberry peel tissue following YY and SQ treatments. Of these, 481 were differentially expressed in both treatments (Figure 4A) (Supplementary Table 2). Of the 481 DEGs, 94 had path annotations. These 94 genes were categorized into seven groups according biological pathways, including carbohydrate metabolism (14 genes, 14.89%), metabolism of other amino acids (14 genes, 14.89%), biosynthesis of other secondary metabolites (11 genes, 11.70%), lipid metabolism (11 genes, 11.70%), metabolism of terpenoids and polyketides (11 genes, 11.70%), environmental adaptation (seven genes, 7.45%), and others (26 genes, 27.66%) (Figure 4B). Further analysis divided the 11 genes implicated in secondary metabolic biosynthesis into four categories: phenylpropanoid biosynthesis (seven genes, 63.64%), isoquinoline alkaloid biosynthesis (two genes, 18.18%), flavonoid biosynthesis (one gene, 9.09%), and anthocyanin biosynthesis (one gene, 9.09%) (Figure 4C). Figure 4D shows the hierarchical clustering of the differentially expressed profiles of genes involved in phenylpropanoid biosynthesis (Supplementary Table 3). The phenylpropanoid biosynthesis pathway encompassed several differentially expressed genes, including five peroxidase genes (g282, g6517, g37110, g46030, and g45681), a probable mannitol dehydrogenase gene (g41069), and a beta-glucosidase 12 gene (g4457). Two differentially expressed genes (DEGs) were downregulated, whereas five DEGs were upregulated. Furthermore, the DEGs that were down-regulated or up-regulated in both treatments were consistent.

#### 3.3.3 Inconsistently DEGs identified by transcriptome analysis treated with KXF6501 fermentation solution and cell-free supernatant

**FIGURE 5:**
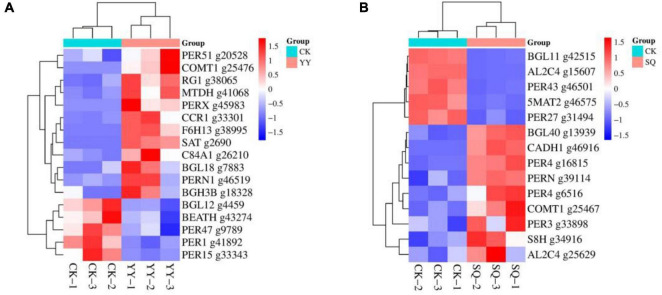
Cluster analysis was conducted on the expression profiles of specific genes associated with phenylpropanoid biosynthesis in CK-vs-YY **(A)** and CK-vs-SQ **(B)**.

Compared with the control, the phenylpropanoid biosynthesis pathway in YY includes the following DEGs: six peroxidase genes (g45983, g46519, g20528, g9789, g41892, and g33343),three beta-glucosidase genes (g18328, g7883, g4459), a Cinnamoyl-CoA reductase gene (g33301), a Raucaffricine-O-beta-D-glucosidase gene (g38065), a Probable mannitol dehydrogenase (g41068), a Stemmadenine O-acetyltransferase gene (g2690), a Caffeic acid 3-O-methyltransferase gene (g25476), a Feruloyl CoA ortho-hydroxylase F6H1-3 (g38995), a Cytochrome P450 84A1 gene (g26210), a Acetyl-CoA-benzylalcohol acetyltransferase (g43274). Among the DEGs (Figure 5A), 12 had upregulated expression while 5 had downregulated expression.

Compared with the control, the phenylpropanoid biosynthesis pathway in SQ exhibits differential expression of several key genes, including six peroxidase genes (g6516, g16815, g33898, g39114, g46501, g31494), two beta-glucosidase genes (g13939, g42515), a Cinnamyl alcohol dehydrogenase gene (g46916), two Aldehyde dehydrogenase genes (g25629, g15607), a Scopoletin 8-hydroxylase gene (g34916), a Caffeic acid 3-O-methyltransferase gene (g25467), and a Pelargonidin 3-O-(6-caffeoylglucoside) 5-O-(6-O-malonylglucoside) malonyltransferase gene (g46575). Nine DEGs were upregulated and five DEGs were downregulated (Figure 5B).

#### 3.3.4 RT-qPCR analysis

**FIGURE 6:**
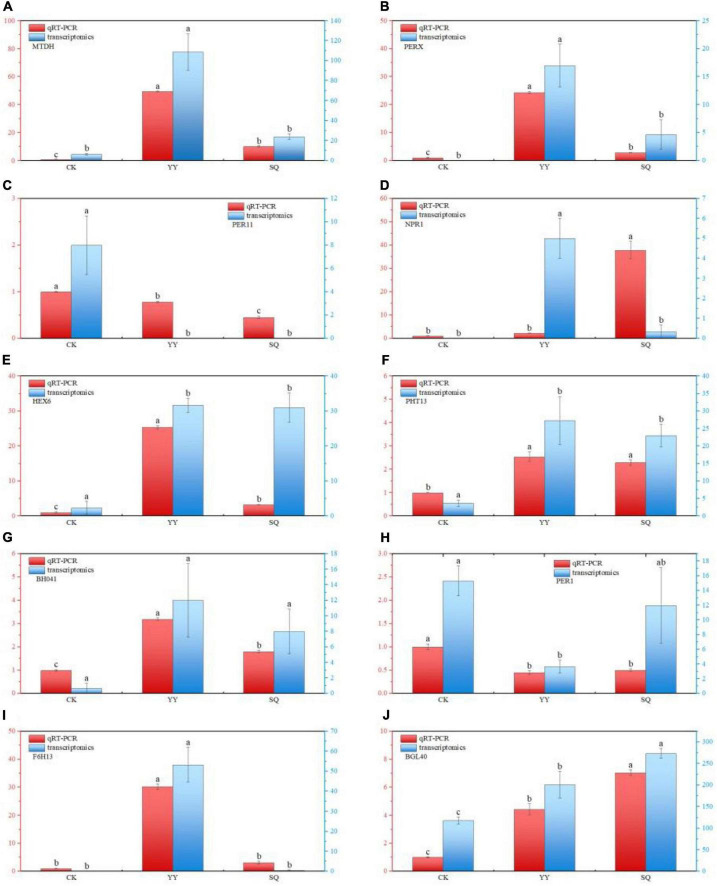
DEGs validation using RT-qPCR for MTDH **(A)**, PERX **(B)**, PER11 **(C)**, NPR1 **(D)**, HEX6 **(E)**, PHT13 **(F)**, BHO41 **(G)**, PER1 (H), F6H13 **(I)**, and BGL40 **(J)**. Based on the Tukey’s multiple range test, there are significant (*P* < 0.05) differences between all variables followed by letters. All datas are reported as means of three distinct values ± SE. Different lowercase letters indicate significant (*P* < 0.05) differences between treatments by the Tukey’s multiple range test.

To assess the credibility of our transcriptome data, we randomly selected ten genes associated with phenylpropane metabolism, transcription factors, and plant hormone signal transduction in fruits. The findings shown in Figure 6 indicate that the expression levels of these genes in the CK, YY, and SQ samples aligned with the transcriptome data, indicating the repeatability and consistency of the RNA-seq data.

### 3.4 Effects of KXF6501 fermentation solution and cell-free supernatant on phenylpropanoid metabolism related enzymes and substances in harvested blueberries

**FIGURE 7:**
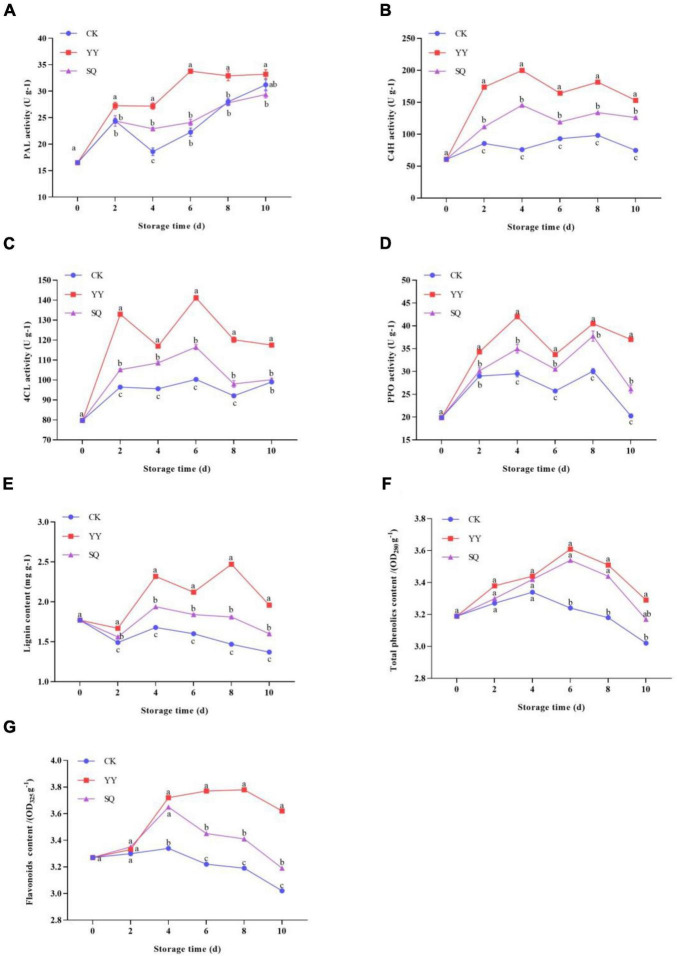
Effects of different treatments (YY and SQ) compared to control (CK) on PAL **(A)**, C4H **(B)**, 4CL **(C)**, PPO **(D)**, lignin content **(E)**, phenol content **(F)**, and flavonoid content **(G)** of blueberries. Different lowercase letters indicate significant (*P* < 0.05) differences between treatments by the Tukey’s multiple range test.

Compared to the control fruits, the application of YY and SQ to blueberry fruits triggered PAL, C4H, 4CL, and PPO activities, as well as an increase in total phenol, flavonoid, and lignin content. PAL activity in all groups exhibited an overall upward trend (Figure 7A), with YY-treated fruits reaching peak activity on the 6th day and displayed significantly higher (*P* < 0.05) PAL activity than that of the control fruits except on the 10th day. SQ-treated fruits maintained higher PAL activity on days 4–10 compared the control. The C4H activity of control fruits increased slightly during the entire storage period (Figure 7B). During storage, the C4H activity was significantly increased by YY and SQ (*P* < 0.05). The peak activity of C4H in YY and SQ fruits was observed 4 d post-treatment, with the enzyme activity being 2.63- and 1.92 times higher (*P* < 0.05) in these fruits than in the control fruits. As shown in Figure 7C, the 4CL enzyme induced by YY showed significantly higher (*P* < 0.05) activity than that induced by SQ and the control during the entire storage period. On the 6th day, the 4CL activity of the YY and SQ treatments reached a peak that was 40.80% and 16.17% higher than that of the control, respectively. All treatment groups showed similar changes in PPO activity (Figure 7D). YY treatment rapidly induced PPO activity, which peak on day 4. All treatments significantly increased PPO activity (*P* < 0.05) during days 6–10, compared with the control. As shown in Figure 7E, YY and SQ notably enhanced lignin levels in blueberry fruits (*P* < 0.05) during days 2–10. The lignin content of YY-treated- and SQ-treated fruits was 67.66% and 22.71% higher, respectively, than that of the control fruits on day 8. The phenol levels of the blueberry fruits across all groups exhibited a similar pattern during storage (Figure 7F). Compared to the control, YY- and SQ-treated fruits had higher levels of total phenols, which increased significantly (*P* < 0.05) during days 6–10. Peak total phenol levels were reached on the 6th day and subsequently declined. The change in flavonoid content (Figure 7G) was consistent with that of total phenols. The YY and SQ treatment groups exhibited significantly higher flavonoid content than in the control fruits on days 4–10 (*P* < 0.05).

### 3.5 Effect of KXF6501 fermentation solution and cell-free supernatant on the postharvest quality of blueberry fruits

As shown in Table 3, the rate of weight loss in the fruits increased with the storage time elongation, with the YY treatment group demonstrating the lowest weight loss rate throughout the storage period, followed by the SQ treatment group. The TSS, TA, AsA and Reducing sugar content in fruits treated with YY were consistently higher than in the control fruits, except at 2 d. Simultaneously, the contents of TSS, TA, AsA and Reducing sugars in fruits treated with SQ in the late storage period (6, 8, and 10 d) were significantly (*P* < 0.05) higher than in the control fruits.

**TABLE 3:** Effect of KXF6501 fermentation solution and cell-free supernatant on the quality parameters of postharvest blueberries.

Quality attribute	Treatment	Storage time (d)				
		**2**	**4**	**6**	**8**	**10**
Weight loss (%)	Control	4.29 ± 0.57a	8.84 ± 1.18a	13.37 ± 1.82a	18.05 ± 2.45a	21.25 ± 2.84a
	YY	2.78 ± 0.24b	7.00 ± 0.68a	11.08 ± 1.17a	14.92 ± 1.51a	17.55 ± 1.72a
	SQ	3.94 ± 0.13a	8.14 ± 0.31a	12.38 ± 0.53a	16.82 ± 0.79a	20.52 ± 1.15a
Total soluble solid (%)	Control	11.07 ± 0.07c	12.27 ± 0.03b	11.97 ± 0.24c	12.60 ± 0.06c	10.20 ± 0.06c
	YY	12.23 ± 0.03b	12.30 ± 0.06b	12.83 ± 0.03b	12.97 ± 0.03b	13.60 ± 0.06a
	SQ	12.87 ± 0.07a	13.93 ± 0.03a	13.37 ± 0.07a	13.70 ± 0.06a	13.33 ± 0.03b
Titrable acid (%)	Control	1.43 ± 0.00a	1.80 ± 0.04a	0.25 ± 0.07b	0.45 ± 0.04b	0.95 ± 0.03a
	YY	0.65 ± 0.05c	1.83 ± 0.05a	0.68 ± 0.04a	1.05 ± 0.00a	0.98 ± 0.04a
	SQ	0.90 ± 0.04b	1.13 ± 0.09b	0.25 ± 0.03b	0.45 ± 0.04b	0.50 ± 0.03b
Ascorbic acid (mg/100g)	Control	52.57 ± 1.19a	34.19 ± 2.47b	22.83 ± 0.43b	22.06 ± 0.79b	26.06 ± 1.37b
	YY	31.75 ± 4.58b	49.02 ± 5.66a	34.01 ± 1.96a	33.26 ± 0.76a	33.80 ± 0.46a
	SQ	33.45 ± 1.19b	33.24 ± 0.95b	36.06 ± 0.97a	34.57 ± 1.23a	40.23 ± 3.32a
Reducing sugar (%)	Control	47.97 ± 0.02b	48.77 ± 0.02c	40.40 ± 0.01c	38.95 ± 0.01c	41.81 ± 0.00c
	YY	44.03 ± 0.01c	48.86 ± 0.01b	45.68 ± 0.01b	56.69 ± 0.02a	54.21 ± 0.00b
	SQ	57.90 ± 0.02a	49.30 ± 0.01a	48.72 ± 0.03a	48.15 ± 0.01b	63.63 ± 0.01a

Based on Tukey’s multiple range test, there were significant (*P* < 0.05) differences between all variables, followed by letters. All data were reported as the mean of three distinct values (SE).

According to Table 4, there is no significant difference in L, a, b, c, and h0 values of blueberries between the YY and SQ treatment groups and the control groups throughout the duration of storage. However, the a/b value over the storage period exhibited a statistically remarkable (*P* < 0.05) increase in the SQ experimental group, with the exception of day 8.

**TABLE 4:** Effects of KXF6501 fermentation solution and cell-free supernatant on color variation in postharvest blueberries.

Color characteristics	Treatment	Storage time (d)				
		**2**	**4**	**6**	**8**	**10**
L	Control	39.36 ± 1.53a	39.39 ± 1.45a	39.55 ± 1.51a	39.45 ± 1.39a	38.31 ± 1.37a
	YY	37.78 ± 1.19a	39.15 ± 1.15a	38.25 ± 1.49a	37.60 ± 1.55a	38.88 ± 0.66a
	SQ	35.76 ± 1.72a	36.70 ± 1.50a	36.28 ± 1.44a	35.68 ± 1.40a	35.83 ± 1.44a
a	Control	−2.99 ± 0.12a	−3.00 ± 0.12ab	−3.01 ± 0.12a	−3.13 ± 0.10a	−2.92 ± 0.11a
	YY	−3.12 ± 0.06a	−3.13 ± 0.04b	−3.20 ± 0.03a	−3.29 ± 0.05a	−3.06 ± 0.03a
	SQ	−2.85 ± 0.09a	−2.81 ± 0.10a	−2.94 ± 0.12a	−3.05 ± 0.10a	−2.80 ± 0.11a
b	Control	−4.92 ± 0.62a	−5.08 ± 0.61a	−5.03 ± 0.64a	−5.04 ± 0.55a	−4.99 ± 0.60a
	YY	−5.02 ± 0.28a	−5.01 ± 0.26a	−5.01 ± 0.29a	−4.87 ± 0.27a	−5.10 ± 0.07a
	SQ	−3.50 ± 0.53a	−3.70 ± 0.46a	−3.75 ± 0.47a	−3.69 ± 0.51a	−3.75 ± 0.44a
a/b	Control	0.56 ± 0.02b	0.55 ± 0.02b	0.56 ± 0.03b	0.58 ± 0.03b	0.55 ± 0.03b
	YY	0.63 ± 0.04b	0.64 ± 0.05b	0.65 ± 0.05b	0.69 ± 0.05ab	0.60 ± 0.01b
	SQ	0.88 ± 0.09a	0.80 ± 0.06a	0.82 ± 0.07a	0.89 ± 0.11a	0.78 ± 0.07a
c	Control	5.79 ± 0.56a	5.93 ± 0.56a	5.89 ± 0.59a	5.96 ± 0.49a	5.81 ± 0.55a
	YY	5.92 ± 0.23a	5.92 ± 0.20a	5.96 ± 0.22a	5.89 ± 0.20a	5.95 ± 0.06a
	SQ	4.55 ± 0.47a	4.66 ± 0.43a	4.78 ± 0.44a	4.83 ± 0.43a	4.71 ± 0.40a
h_0_	Control	237.03 ± 3.59a	238.07 ± 3.07a	237.43 ± 3.47a	236.92 ± 3.02a	238.22 ± 3.17a
	YY	237.69 ± 1.69a	237.59 ± 1.75a	237.10 ± 2.01a	235.63 ± 1.88a	238.90 ± 0.38a
	SQ	229.31 ± 3.06a	231.72 ± 2.36a	230.67 ± 2.31a	229.10 ± 3.42a	232.36 ± 2.50a

Based on Tukey’s multiple range test, there were significant (*P* < 0.05) differences between all variables, followed by letters. All data were reported as the mean of three distinct values (SE).

## 4 Discussion

Post-harvest blueberry fruits are easily infected with *B. cinerea* during storage and transportation, leading to the decay of blueberry fruits, shortening their storage period, and affecting their flavor and nutritional value (Bell et al., 2021). Therefore, decay emerged as the predominant factor influencing blueberry quality during storage, necessitating the development of treatment strategies to prolong storage life (Ding et al., 2023). In recent years, microbial antagonists derived from the *Bacillus* genus have been recognized as environmentally friendly and biologically safe alternatives to conventional chemical fungicides (Lastochkina et al., 2019). The use of *Bacillus* spp. as biocontrol formulations to mitigate in vegetables and fruits postharvest diseases has gained increasing attention (Koh et al., 2014; Arroyave-Toro et al., 2017). The *B. velezensis* SL-6 strain has an inhibitory effect on *B. cinerea* in pears and can significantly reduce the incidence of *B. cinerea* and the expansion of lesion diameter during pear storage (Cozzolino et al., 2020). *Bacillus* strains M9-20 decreased the growth of *B. cinerea* spores and mycelia, inhibited blueberry fruit rot caused by pathogenic bacteria, and reduced the incidence and severity of rot by 50% (Kurniawan et al., 2018). *Bacillus* spp. are recognized as safe microorganisms suitable for the food industry (Algburi et al., 2016), and some *Bacillus* strains are recognized as probiotic bacteria (Khochamit et al., 2015). In this study, a fermentation solution (YY) of the BCA *B. tequilensis* KXF6501 which was isolated from healthy strawberries, significantly inhibited spore germination and reduced germ tube length in *B. cinerea*, followed by the cell-free supernatant (SQ). We also analyzed the antagonistic effects of KXF6501 (YY and SQ) on *B. cinerea* mycelium growth *in vitro*. Indicating that both YY and SQ exhibited inhibitory effects, with YY demonstrating a greater efficacy against *B. cinerea* than SQ. The YY and SQ treatments reduced the incidence of fruits and lesion diameter, thus effectively controlling the gray mold of blueberries *in vivo*. The control effect of YY was better than that of SQ. This may be due to competition for nutrients and space between microbial antagonists and phytopathogenic microorganisms (Carmona-Hernandez et al., 2019; Bell et al., 2021). At the same time, the attachment of microbial antagonists to the hyphae of pathogens significantly slows pathogen growth and spore germination, as antagonists have a higher nutrient absorption rate than the target pathogens (Sharma et al., 2009; Singh et al., 2022). Moreover, certain antifungal compounds may be essential for protection against pathogenic infections (Koh et al., 2014; Arroyave-Toro et al., 2017).

The biological control mechanism of *Bacillus* spp. involves the production of antimicrobial compounds that hinder the growth of plant pathogens (Guardado-Valdivia et al., 2018). Little is known, nevertheless, about the basic process of fruit disease resistance induction, which is limited to a single metabolite, enzyme, or gene (Lu et al., 2015). We conducted an analysis of the transcriptome of fruits treated with YY and SQ to further study the possible defense responses of fruits induced by YY and SQ using Illumina sequencing technology. The results showed that YY and SQ caused substantial changes in mRNAs levels compared to the control fruits (Figures 2B–D). Furthermore, according to the biological pathway enrichment analysis, the blueberries resistance response to YY and SQ was influenced by the biosynthesis of secondary metabolites (Figure 3). These findings are consistent with the results obtained from the analysis of biological pathways that included consistently differentially expressed genes (DEGs) in both treatments (Figure 4B). Simultaneously, the consistent DEGs linked to secondary metabolite biosynthesis underscore the significance of phenylpropanoid biosynthesis as a key secondary metabolic process in fruit resistance induction (Figure 4). The proportion of consistent DEGs involved in phenylpropanoid biosynthesis was the highest, displaying consistent expression patterns across both treatments. In particular, the expression levels of cationic peroxidase 1, probable mannitol dehydrogenase, lignin-forming anionic peroxidase, peroxidase 4, and peroxidase 12, all of which play roles in the synthesis of crucial enzymes, were elevated in fruits treated with YY and SQ compared to those in control fruits. In addition, we analyzed the inconsistent DEGs related to phenylpropane metabolism in the YY and SQ treatments. In the YY treatment group, 12 DEGs were downregulated, and 12 DEGs were upregulated. Similarly, in the SQ treatment group, 5 DEGs were downregulated, whereas 9 DEGs were upregulated. The majority of Differentially Expressed Genes (DEGs) are actively involved in critical enzymes PAL, POD, C4H, and 4CL. This finding aligns with the results studied by Lu et al. (2015), who discovered the upregulation of critical genes related to phenylpropanoid metabolism and biosynthesis in yeast-treated fruits.

The phenylpropanoid pathway is one of important plant secondary metabolism, and is beneficial for wound healing and resistance enhancement in fruits and vegetables (Wang B. et al., 2019). The primary products of this pathway include antifungal plant antitoxins, phenolic acids (associated with the biosynthesis of secondary cell walls), lignin, and essential precursors like chalcones that serve as initiators of the flavonoid pathway (Tan et al., 2016; Zhou et al., 2018). Antifungal plant antitoxins, phenolic acids (associated with the biosynthesis of secondary cell walls), lignin, and essential precursors like chalcones (initiating flavonoid biosynthesis) are the primary products of this pathway. Lignin primarily consists of phenylpropanoid units resulting from the oxidative polymerization of hydroxycinnamoyl alcohol derivatives. Its biosynthesis plays a crucial role in enhancing the structural integrity of the cell wall, and serves as a physical barrier against pathogens and their reproduction (Ferrer et al., 2008; Liu et al., 2014; Zhou et al., 2018). Vital enzymes such as PAL, C4H, and 4CL in the phenylpropanoid pathway are essential for regulating the smooth progression of this biosynthetic pathway (Muro-Villanueva et al., 2019). PAL facilitates the transformation of l-phenylalanine into trans-cinnamic acid, followed by cinnamate C4H, which catalyzes the transformation of trans-cinnamic acid into hydroxycinnamic acid to produce lignin biosynthesis precursors, including coumaric acid, ferulic acid, and caffeic acid. Subsequently, 4CL catalyzes the synthesis of coenzyme A thioesters, which are converted to flavonoids. Simultaneously, p-coumaroyl coenzyme A is catalyzed by lignin (Du et al., 2023). In addition, phenols are oxidized to quinones by PPO, which are directly toxic to pathogens (Shadle et al., 2003). Flavonoids exhibit antioxidant properties and anti-bacterial action, such as the inhibition of spore germination and mycelium growth (Liu et al., 2014).

To verify the upregulation of genes in the phenylpropane pathway at the biochemical level, we measured enzyme activity (PAL, C4H, 4CL, and PPO) and the content of three metabolites (flavonoids, total phenols and lignin) at various time points in blueberry peel. The findings showed that treatment with YY and SQ significantly increased the activities of the defense-related enzymes PAL, C4H, 4CL, and PPO and promoted the accumulation of total phenols, flavonoids, and lignin in fruits. By comparing with the treatment with SQ, YY proved to be more effective in accumulating higher levels of enzyme activities and substance related to the phenylpropane pathway. This may be attributed to the fact that in addition to the antimicrobial substances in the supernatant, cells in *B. tequilensis* fermentation broth also play an important role (Carmona-Hernandez et al., 2019). The *Bacillus* cells colonize the surface of the fruit, which act as inducers to stimulate the enzymes activity and substances related to the phenylpropanoid metabolism pathway, thus protecting the fruit from pathogen attack. The accumulation of lignin, flavonoids, and total phenols in postharvest fruits following treatment with *Bacillus* may be attributed to the upregulation of key enzymes in the phenylpropanoid pathway (Wang et al., 2023). Moreover, postharvest blueberry fruits treated with YY and SQ exhibited significantly stable quality during storage compared to the control groups, highlighting the potential of KXF6501 to preserve the appearance and nutritional quality of postharvest blueberry fruits. These results show that the BCA of *B. tequilensis* KXF6501 can serve as a viable approach for mitigating postharvest fruit diseases.

## 5 Conclusion

*B. tequilensis* KXF6501 can reduce incidence and lesion diameter caused by *B. cinerea gray* mold during blueberries storage, thereby preserving postharvest fruit quality. Application of *B. tequilensis* KXF6501 (YY and SQ) resulted in significant upregulation of genes associated with phenylpropanoid biosynthesis, leading to the elevated enzyme activities and accumulation of antifungal compounds in phenylpropanoid pathway. That enhanced disease resistance in blueberries. When compared to the SQ treatment, YY proved to be more effective in controlling gray mold disease by accumulating higher levels of enzyme activity as well as substances related to the phenylpropane pathway. Therefore, *B. tequilensis* KXF6501 may be a potential BCA for blueberry gray mold. However, we need to further explore the mechanism of action of *B. tequilensis* KXF6501 in controlling postharvest diseases of fruits for assessing the safety of both *B. tequilensis* KXF6501 and its metabolites on consumer health. These results of this research offer insights into the use of *B. tequilensis* KXF6501 for the biological management of postharvest diseases in blueberries.

## Data Availability

The datasets presented in this study can be found in online repositories. The names of the repository/repositories and accession number(s) can be found in this article/Supplementary material.

## Footnotes

1
http://www.geneontology.org/

2
http://www.genome.jp/kegg/

## References

[B1] Algburi A. Volski A. Cugini C. Walsh E. Chistyakov V. Mazanko M. (2016). Safety properties and probiotic potential of *Bacillus subtilis* KATMIRA1933 and *Bacillus amyloliquefaciens* B-1895. *Adv. Microbiol.* 6 432–452. 10.4236/aim.2016.66043

[B2] Alvarez A. Gelezoglo R. Garmendia G. González M. L. Magnoli A. P. Arrarte E. (2019). Role of Antarctic yeast in biocontrol of *Penicillium expansum* and patulin reduction of apples. *Environ. Sustain.* 2 277–283. 10.1007/s42398-019-00081-1

[B3] Arroyave-Toro J. J. Mosquera S. Villegas-Escobar V. (2017). Biocontrol activity of *Bacillus subtilis* EA-CB0015 cells and lipopeptides against postharvest fungal pathogens. *Biol. Control* 114 195–200. 10.1016/j.biocontrol.2017.08.014

[B4] Bell S. R. Hernández Montiel L. G. González Estrada R. R. Gutiérrez Martínez P. (2021). Main diseases in postharvest blueberries, conventional and eco-friendly control methods: A review. *LWT* 149:112046. 10.1016/j.lwt.2021.112046

[B5] Bi K. Liang Y. Mengiste T. Sharon A. (2023). Killing softly: A roadmap of *Botrytis cinerea* pathogenicity. *Trends Plant Sci.* 28 211–222. 10.1016/j.tplants.2022.08.024 36184487

[B6] Carmona-Hernandez S. Reyes-Pérez J. J. Chiquito-Contreras R. G. Rincon-Enriquez G. Cerdan-Cabrera C. R. Hernandez-Montiel L. G. (2019). Biocontrol of postharvest fruit fungal diseases by bacterial antagonists: A review. *Agronomy* 9:121. 10.3390/agronomy9030121

[B7] Chen H. Cao S. Fang X. Mu H. Yang H. Wang X. (2015). Changes in fruit firmness, cell wall composition and cell wall degrading enzymes in postharvest blueberries during storage. *Sci. Horticult.* 188 44–48. 10.1016/j.scienta.2015.03.018

[B8] Chen K. Tian Z. He H. Long C.-A. Jiang F. (2020). *Bacillus* species as potential biocontrol agents against citrus diseases. *Biol. Control* 151:104419. 10.1016/j.biocontrol.2020.104419

[B9] Chen Y. Hung Y.-C. Chen M. Lin H. (2017). Effects of acidic electrolyzed oxidizing water on retarding cell wall degradation and delaying softening of blueberries during postharvest storage. *LWT* 84 650–657. 10.1016/j.lwt.2017.06.011

[B10] Cozzolino M. E. Distel J. S. García P. A. Mascotti M. L. Ayub M. J. Benazzi L. M. (2020). Control of postharvest fungal pathogens in pome fruits by lipopeptides from a *Bacillus* sp. isolate SL-6. *Sci. Horticult.* 261:108957. 10.1016/j.scienta.2019.108957

[B11] Deng L. Yin B. Yao S. Wang W. Zeng K. (2016). Postharvest application of oligochitosan and chitosan reduces calyx alterations of citrus fruit induced by ethephon degreening treatment. *J. Agric. Food Chem.* 64 7394–7403. 10.1021/acs.jafc.6b02534 27618996

[B12] Ding J. Liu C. Huang P. Zhang Y. Hu X. Li H. (2023). Effects of thymol concentration on postharvest diseases and quality of blueberry fruit. *Food Chem.* 402:134227. 10.1016/j.foodchem.2022.134227 36155290

[B13] Du R. Deng J. Huang E. Chen L. Tang J. Liu Y. (2023). Effects of salicylic acid-grafted bamboo hemicellulose on gray mold control in blueberry fruit: The phenylpropanoid pathway and peel microbial community composition. *Int. J. Biol. Macromol.* 251:126303. 10.1016/j.ijbiomac.2023.126303 37573915

[B14] Ferrer J. L. Austin M. B. Stewart C. Noel J. P. (2008). Structure and function of enzymes involved in the biosynthesis of phenylpropanoids. *Plant Physiol. Biochem.* 46 356–370. 10.1016/j.plaphy.2007.12.009 18272377 PMC2860624

[B15] Guardado-Valdivia L. Tovar-Pérez E. Chacón-López A. López-García U. Gutiérrez-Martínez P. Stoll A. (2018). Identification and characterization of a new *Bacillus atrophaeus* strain B5 as biocontrol agent of postharvest anthracnose disease in soursop (*Annona muricata*) and avocado (*Persea americana*). *Microbiol. Res.* 210 26–32. 10.1016/j.micres.2018.01.007 29625655

[B16] Ji Y. Hu W. Jiang A. Xiu Z. Liao J. Yang X. (2019). Effect of ethanol treatment on the quality and volatiles production of blueberries after harvest. *J. Sci. Food Agric.* 99 6296–6306. 10.1002/jsfa.9904 31260121

[B17] Khochamit N. Siripornadulsil S. Sukon P. Siripornadulsil W. (2015). Antibacterial activity and genotypic–phenotypic characteristics of bacteriocin-producing *Bacillus subtilis* KKU213: Potential as a probiotic strain. *Microbiol. Res.* 170 36–50. 10.1016/j.micres.2014.09.004 25440998

[B18] Kiesewalter H. T. Lozano-Andrade C. N. Maróti G. Snyder D. Cooper V. S. Jørgensen T. S. (2020). Complete Genome sequences of 13 *Bacillus subtilis* soil isolates for studying secondary metabolite diversity. *Microbiol. Resour. Announc.* 9 10–1128. 10.1128/mra.01406-19 31919181 PMC6952667

[B19] Koh Y. Jung J. Hur J. Kim G. (2014). Control of postharvest fruit rot diseases of kiwifruit by antagonistic bacterium *Bacillus subtilis*. *Int. Symp. Kiwifruit* 1096 377–382. 10.17660/ActaHortic.2015.1096.44

[B20] Kurniawan O. Wilson K. Mohamed R. Avis T. J. (2018). Bacillus and *Pseudomonas* spp. provide antifungal activity against gray mold and Alternaria rot on blueberry fruit. *Biol. Control* 126 136–141. 10.1016/j.biocontrol.2018.08.001

[B21] Lastochkina O. Seifikalhor M. Aliniaeifard S. Baymiev A. Pusenkova L. Garipova S. (2019). *Bacillus* Spp.: Efficient biotic strategy to control postharvest diseases of fruits and vegetables. *Plants* 8:97. 10.3390/plants8040097 31013814 PMC6524353

[B22] Liu Y. Ge Y. Bi Y. Li C. Deng H. Hu L. (2014). Effect of postharvest acibenzolar-S-methyl dipping on phenylpropanoid pathway metabolism in muskmelon (*Cucumis melo* L.) fruits. *Sci. Horticult.* 168 113–119. 10.1016/j.scienta.2014.01.030

[B23] Lu L. Ji L. Shi R. Li S. Zhang X. Guo Q. (2019). Dextran as an elicitor of phenylpropanoid and flavonoid biosynthesis in tomato fruit against gray mold infection. *Carbohydr. Polym.* 225:115236. 10.1016/j.carbpol.2019.115236 31521274

[B24] Lu L. Wang J. Zhu R. Lu H. Zheng X. Yu T. (2015). Transcript profiling analysis of *Rhodosporidium paludigenum*-mediated signalling pathways and defense responses in mandarin orange. *Food Chem.* 172 603–612. 10.1016/j.foodchem.2014.09.097 25442597

[B25] Lu Y. Ma D. He X. Wang F. Wu J. Liu Y. (2021). *Bacillus subtilis* KLBC BS6 induces resistance and defence-related response against *Botrytis cinerea* in blueberry fruit. *Physiol. Mol. Plant Pathol.* 114:101599. 10.1016/j.pmpp.2020.101599

[B26] Miller K. Feucht W. Schmid M. (2019). Bioactive compounds of strawberry and blueberry and their potential health effects based on human intervention studies: A brief overview. *Nutrients* 11:1510. 10.3390/nu11071510 31269727 PMC6683271

[B27] Muro-Villanueva F. Mao X. Chapple C. (2019). Linking phenylpropanoid metabolism, lignin deposition, and plant growth inhibition. *Curr. Opin. Biotechnol.* 56 202–208. 10.1016/j.copbio.2018.12.008 30677701

[B28] Ozgen M. Scheerens J. Reese N. Miller R. (2010). Total phenolic, anthocyanin contents and antioxidant capacity of selected elderberry (*Sambucus canadensis* L.) accessions. *Pharmacogn. Magaz.* 6:198. 10.4103/0973-1296.66936 20931079 PMC2950382

[B29] Panebianco S. Vitale A. Polizzi G. Scala F. Cirvilleri G. (2015). Enhanced control of postharvest citrus fruit decay by means of the combined use of compatible biocontrol agents. *Biol. Control* 84 19–27. 10.1016/j.biocontrol.2015.02.001

[B30] Papoutsis K. Mathioudakis M. M. Hasperué J. H. Ziogas V. (2019). Non-chemical treatments for preventing the postharvest fungal rotting of citrus caused by *Penicillium digitatum* (green mold) and *Penicillium italicum* (blue mold). *Trends Food Sci. Technol.* 86 479–491. 10.1016/j.tifs.2019.02.053

[B31] Rivera S. A. Zoffoli J. P. Latorre B. A. (2013). Determination of optimal sulfur dioxide time and concentration product for postharvest control of gray mold of blueberry fruit. *Postharvest Biol. Technol.* 83 40–46. 10.1016/j.postharvbio.2013.03.007

[B32] Shadle G. L. Wesley S. V. Korth K. L. Chen F. Lamb C. Dixon R. A. J. P. (2003). Phenylpropanoid compounds and disease resistance in transgenic tobacco with altered expression of L-phenylalanine ammonia-lyase. *Phytochemistry* 64 153–161. 10.1016/S0031-9422(03)00151-1 12946414

[B33] Sharma R. Singh D. Singh R. (2009). Biological control of postharvest diseases of fruits and vegetables by microbial antagonists: A review. *Biol. Control* 50 205–221. 10.1016/j.biocontrol.2009.05.001

[B34] Shi C. Yan P. Li J. Wu H. Li Q. Guan S. (2014). Biocontrol of *Fusarium graminearum* growth and deoxynivalenol production in wheat kernels with bacterial antagonists. *Int. J. Environ. Res. Public Health* 11 1094–1105. 10.3390/ijerph110101094 24441510 PMC3924494

[B35] Shi Z. Deng J. Wang F. Liu Y. Jiao J. Wang L. (2019a). Individual and combined effects of bamboo vinegar and peach gum on postharvest grey mould caused by *Botrytis cinerea* in blueberry. *Postharvest Biol. Technol.* 155 86–93. 10.1016/j.postharvbio.2019.05.016

[B36] Shi Z. Yang H. Jiao J. Wang F. Lu Y. Deng J. (2019b). Effects of graft copolymer of chitosan and salicylic acid on reducing rot of postharvest fruit and retarding cell wall degradation in grapefruit during storage. *Food Chem.* 283 92–100. 10.1016/j.foodchem.2018.12.078 30722930

[B37] Shi Z. Wang F. Lu Y. Deng J. (2018). Combination of chitosan and salicylic acid to control postharvest green mold caused by *Penicillium digitatum* in grapefruit fruit. *Sci. Horticult.* 233 54–60. 10.1016/j.scienta.2018.01.039

[B38] Singh A. K. Sharma P. (2020). Disinfectant-like activity of lipopeptide biosurfactant produced by *Bacillus tequilensis* strain SDS21. *Colloids Surf. B Biointerf.* 185:110514. 10.1016/j.colsurfb.2019.110514 31639569

[B39] Singh S. K. Singh V. K. Singh P. K. Modi A. Kumar A. (2022). Microbial antagonists in postharvest management of fruit. *Res. Technol. Adv. Food Sci.* 2022 333–346. 10.1016/B978-0-12-824369-5.00005-1

[B40] Tan B. A. Daim L. D. J. Ithnin N. Ooi T. E. K. Md-Noh N. Mohamed M. (2016). Expression of phenylpropanoid and flavonoid pathway genes in oil palm roots during infection by *Ganoderma boninense*. *Plant Gene* 7 11–20. 10.1016/j.plgene.2016.07.003

[B41] Toscano S. Ferrante A. Leonardi C. Romano D. (2018). PAL activities in asparagus spears during storage after ammonium sulfate treatments. *Postharvest Biol. Technol.* 140 34–41. 10.1016/j.postharvbio.2018.02.010

[B42] Wang B. Jiang H. Bi Y. He X. Wang Y. Li Y. (2019). Preharvest multiple sprays with sodium nitroprusside promote wound healing of harvested muskmelons by activation of phenylpropanoid metabolism. *Postharvest Biol. Technol.* 158:110988. 10.1016/j.postharvbio.2019.110988

[B43] Wang F. Deng J. Jiao J. Lu Y. Yang L. Shi Z. (2019). The combined effects of Carboxymethyl chitosan and *Cryptococcus laurentii* treatment on postharvest blue mold caused by *Penicillium italicum* in grapefruit fruit. *Sci. Horticult.* 253 35–41. 10.1016/j.scienta.2019.04.031

[B44] Wang R. Zhang Q. Li B. Weng Q. Liu P. (2024). Dimethyl trisulfide (DMTS) fumigation enhances fruit quality and reduces postharvest incidence of bacterial canker in tomato. *Postharvest Biol. Technol.* 216:113038. 10.1016/j.postharvbio.2024.113038

[B45] Wang X. Xie S. Mu X. Guan B. Hu Y. Ni Y. (2023). Investigating the resistance responses to *Alternaria brassicicola* in ‘Korla’ fragrant pear fruit induced by a biocontrol strain *Bacillus subtilis* Y2. *Postharvest Biol. Technol.* 199:112293. 10.1016/j.postharvbio.2023.112293

[B46] Wu C. Wang Y. Ai D. Li Z. Wang Y. (2022). Biocontrol yeast T-2 improves the postharvest disease resistance of grape by stimulation of the antioxidant system. *Food Sci. Nutr.* 10 3219–3229. 10.1002/fsn3.2940 36249987 PMC9548374

[B47] Yihui G. Song J. Du L. Vinqvist M. Palmer L. C. Fillmore S. (2018). Characterization of laccase from apple fruit during postharvest storage and its response to diphenylamine and 1-methylcyclopropene treatments. *Food Chem.* 253 314–321. 10.1016/j.foodchem.2018.01.142 29502838

[B48] Zhang L. Wang L. Zeng X. Chen R. Yang S. Pan S. (2019). Comparative transcriptome analysis reveals fruit discoloration mechanisms in postharvest strawberries in response to high ambient temperature. *Food Chem. X* 2:100025. 10.1016/j.fochx.2019.100025 31432012 PMC6694852

[B49] Zhou H. Zhu H. Ren Z. Li X. Zhong J. Liu E. (2021). Efficacy of *Bacillus tequilensis* strain JN-369 to biocontrol of rice blast and enhance rice growth. *Biol. Control* 160:104652. 10.1016/j.biocontrol.2021.104652

[B50] Zhou Y. Ma J. Xie J. Deng L. Yao S. Zeng K. (2018). Transcriptomic and biochemical analysis of highlighted induction of phenylpropanoid pathway metabolism of citrus fruit in response to salicylic acid, *Pichia membranaefaciens* and oligochitosan. *Postharvest Biol. Technol.* 142 81–92. 10.1016/j.postharvbio.2018.01.021

[B51] Zhou Y. Ming J. Deng L. Zeng K. (2014). Effect of *Pichia membranaefaciens* in combination with salicylic acid on postharvest blue and green mold decay in citrus fruits. *Biol. Control* 74 21–29. 10.1016/j.biocontrol.2014.03.007

