# Supplementary material for: Application of Bacillus tequilensis for the control of gray mold caused by Botrytis cinerea in blueberry and mechanisms of action: inducing phenylpropanoid pathway metabolism
Source: Front Microbiol. 2024 Aug 30;15:1455008. doi: 10.3389/fmicb.2024.1455008 (PMC11392732; doi:10.3389/fmicb.2024.1455008)
Supplement: Supplementary file 1 [file Table_1.DOCX]

Supplementary Table1 Specific primers sequences for differentially expressed genes (DEGs)

| Primer**名称** | **序列(5' to 3')** |
| --- | --- |
| MTDH-g 41069-Left | GGTGACTCAAGTGGGATGCA |
| MTDH-g 41069-Right | TAACGGCTTTGGGGCAGTAG |
| PERX-g 37110-Left | ACCACAGCCAACGTTGATCT |
| PERX-g 37110-Right | ACTAAGACCCTGCCTAGCGA |
| PER11-g 45681-Left | CATACAATCGGCATGGCACG |
| PER11-g 45681-Right | GCAAGTTGCTCTCAGGTTGC |
| PER1-g 41892-Left | GCGAACGCAGACATTCCATC |
| PER1-g 41892-Right | GCACCTTGCAAGACCAATGG |
| BGL40-g 13939-Left | ACTAGCCTGAATCCGGGGAT |
| BGL40-g 13939-Right | GATCCGAGCCGCCAAGATAA |
| F6H13-g 38995-Left | TCACGGTGTCCCCATTGAAG |
| F6H13-g 38995-Right | GCCGTAGCGCACATTGTTAG |
| PHT13-g 31797-Left | ACTTAGGTCCACTTGCCACG |
| PHT13-g 31797-Right | GGGCTCGGACGAGTACAAAA |
| NPR1-g 22602-Left | CGAGCCGTTGATTACCGAGA |
| NPR1-g 22602-Right | CAACACTGGAGCTAGGCACA |
| BH041-g 22595-Left | TTCCTCCCTCCTAGAGCCAC |
| BH041-g 22595-Right | CTTCTGTCGTTGTCGCCTCT |
| HEX6-g 16607-Left | GATTCCAATTCAGCGTCGGC |
| HEX6-g 16607-Right | AGGATCAAAGCAGGGACAGC |
| GAPDH-Left (内参) | GCTGTACCACAAACTGTCTTGC |
| GAPDH-Right (内参) | ATGAAGCAGCTCTTCCACCTCT |
