# Supplementary material for: Application of Bacillus tequilensis for the control of gray mold caused by Botrytis cinerea in blueberry and mechanisms of action: inducing phenylpropanoid pathway metabolism
Source: Front Microbiol. 2024 Aug 30;15:1455008. doi: 10.3389/fmicb.2024.1455008 (PMC11392732; doi:10.3389/fmicb.2024.1455008)
Supplement: Supplementary file 3 [file Table_3.DOCX]

Supplementary Table3 phenylpropanoid biosynthesis-related DEGs in both treatments YY and SQ

| Gene ID |
| --- |
| Vadar_g282_Vaccinium_darrowii_v1.2 |
| Vadar_g41069_Vaccinium_darrowii_v1.2 |
| Vadar_g6517_Vaccinium_darrowii_v1.2 |
| Vadar_g37110_Vaccinium_darrowii_v1.2 |
| Vadar_g46030_Vaccinium_darrowii_v1.2 |
| Vadar_g4457_Vaccinium_darrowii_v1.2 |
| Vadar_g45681_Vaccinium_darrowii_v1.2 |
