# Supplementary figures and images for: Application of Bacillus tequilensis for the control of gray mold caused by Botrytis cinerea in blueberry and mechanisms of action: inducing phenylpropanoid pathway metabolism
Source: Front Microbiol. 2024 Aug 30;15:1455008. doi: 10.3389/fmicb.2024.1455008 (PMC11392732; doi:10.3389/fmicb.2024.1455008)

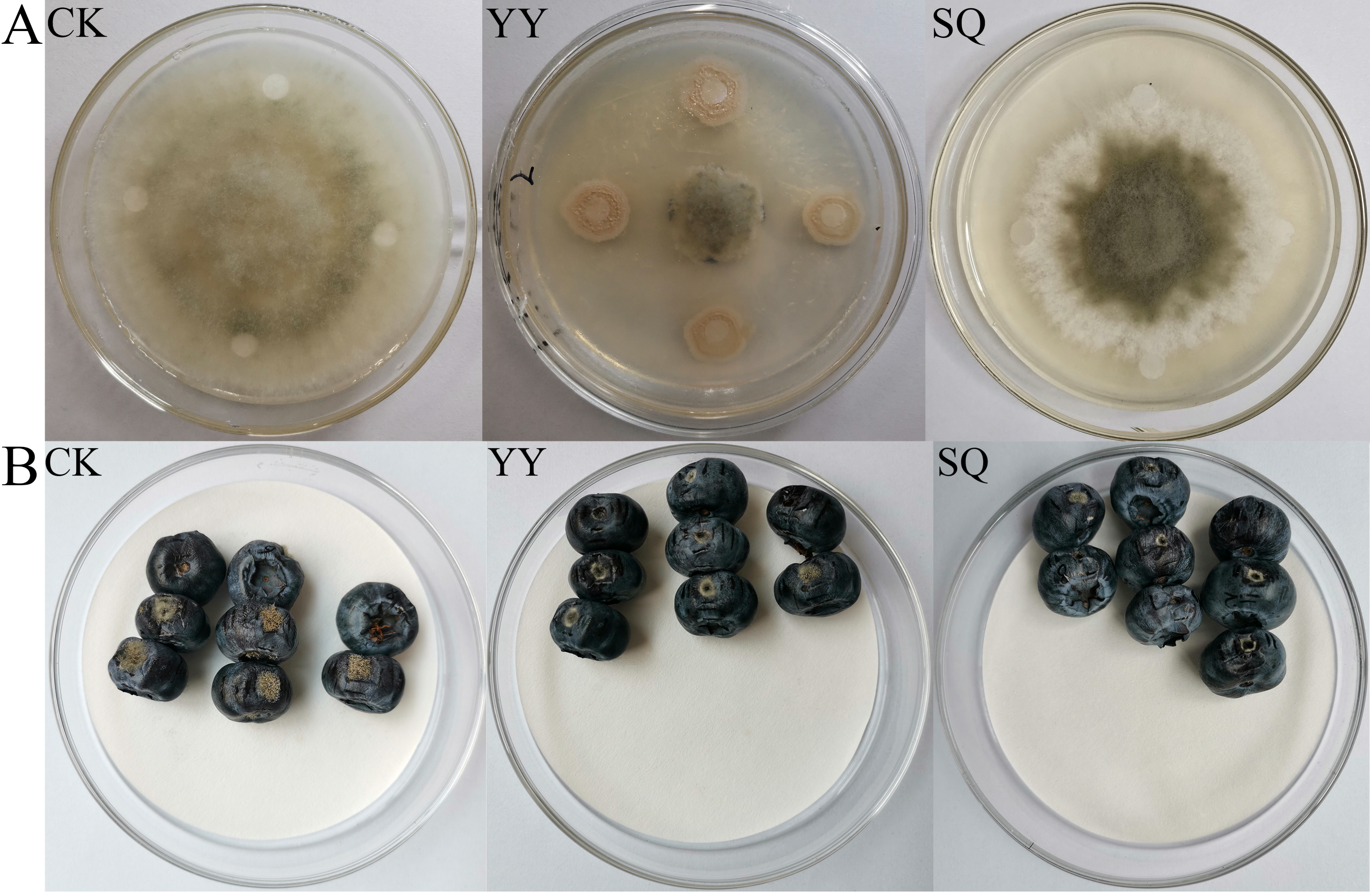

Supplement: Supplementary file 4 [file Image_1.tif]
